# A fatal case of oxygen embolism in a hospital

**DOI:** 10.1080/20961790.2017.1329695

**Published:** 2017-05-31

**Authors:** Lionel Comment, Vincent Varlet, Kewin Ducrot, Silke Grabherr

**Affiliations:** a Forensic Medicine Unit, University Center of Legal Medicine, CHUV Hospital, Lausanne-Geneva, Switzerland; b Forensic Toxicology and Chemistry Unit, University Center of Legal Medicine, CHUV Hospital, Lausanne-Geneva, Switzerland; c Unit of Forensic Imaging and Anthropology, University Center of Legal Medicine, CHUV Hospital, Lausanne-Geneva, Switzerland

**Keywords:** Forensic science, air embolism, autopsy, forensic imaging, gas analysis

## Abstract

This case reports on a 68-year-old man who was found dead in hospital next to his bed. Before this, he had been treated with intravenous antibiotics for pneumonia. The body was found with a peripheral venous catheter connected to a nasal cannula delivering oxygen (O_2_) from the wall.

Extensive medico–legal examinations were performed, including post-mortem computed tomography (CT), complete conventional autopsy, histological and immunohistochemistry analysis, toxicological analysis and post-mortem chemistry. Additionally, CT-guided gas sampling was performed at multiple sites to collect samples for gas analysis.

During the external examination, massive subcutaneous emphysema was visible over the entire surface of the body. The CT scan revealed the presence of gas throughout the vascular system, and in the subcutaneous and muscular tissues. The autopsy confirmed the presence of lobar pneumonia and multiple gas bubbles in the vascular system.

The gas analysis results showed a subnormal concentration of oxygen, confirming the suspected pure O_2_ embolism. Moreover, the carbon dioxide (CO_2_) concentration in the gas sample from the heart was elevated to a level similar to those found in scuba diving fatalities. This could come from degassing of dissolved CO_2_ that accumulated and was trapped in the cardiac cavity. Based on the results of the different exams performed, and especially the gas analysis results, it was concluded that the cause of death was O_2_ embolism.

## Introduction

In addition to autopsy, many tools can be used by a forensic pathologist to determine the cause of death. These tools include post-mortem imaging, toxicological analysis, post-mortem chemistry, histology and immunohistochemistry. Although none of these analyses can replace autopsy, they all can help to discriminate between several possible causes of death and confirm the results obtained from other techniques. When available, the circumstances of the death, and the medical and family history of the individual can help the forensic pathologist to perform the autopsy and choose the most useful complementary exams.

Nowadays, post-mortem imaging is very well developed [1,2], and a contrast agent can be used to obtain a very detailed view of the vascular system [3,4]. Gas accumulated in the body can be precisely located and quantified using post-mortem imaging [5], and an assessment of any post-mortem changes [6] can be made by a forensic radiologist to differentiate between an exogenous or an endogenous (i.e. putrefactive) origin of the gas [7]. Analysis of the gas to determine its composition can confirm the imaging findings and help to differentiate between endogenous and exogenous origins [8]. Gas analysis results can indicate if the gas is from air embolism or physiological changes occurring during the perimortem phase [9,10].

At the University Center of Legal Medicine in Lausanne, Switzerland, autopsies are performed at the request of a prosecutor. A native computed tomography (CT) scan is routinely performed before the autopsy, and, in the case of a suspected vascular lesion, a multi-phase post-mortem CT angiography is performed [11]. Samples of major organs (brain, heart, lungs, liver and kidneys) are taken during the autopsy for histological analysis [12]. Multiple samples are taken for toxicological analysis, post-mortem chemistry and genetic analysis. In some specific cases involving gas poisoning, or if unexpected accumulations of gas are identified after the native CT scan, gaseous samples are also taken according to the protocol proposed by Varlet et al. [7].

The present case describes a fatality following a (probable) suicidal oxygen (O_2_) embolism at a hospital and demonstrates the usefulness of gas sampling and analysis for precise diagnosis of gas embolism. To the best of our knowledge, this is the first case described in the literature of CT-guided gas sampling and analysis for a fatal O_2_ embolism.

## Case report

A 68-year-old male was admitted to hospital with a history of dyspnea at rest for 2 d, a cough producing mucus, loss of appetite and diarrhoea. Pneumonia was suspected, and the patient received intravenous antibiotic treatment. During the patient's second night in hospital, he was found groggy and walking near his room by the nurses. They took him back to his room and connected a new antibiotic dose to his venous catheter. A few hours later, he was found unconscious, lying on the floor next to his bed. His peripheral venous catheter was connected to a nasal cannula delivering O_2_ from the wall, with a flow rate of approximately 2 L/min, and the antibiotic dose was found in the garbage can. As post-mortem lividity was present, no resuscitation attempts were performed by the clinicians and the death was pronounced. The body was immediately brought to the University Center of Legal Medicine (Lausanne, Switzerland).

### CT imaging and gas sampling

Before any manipulation of the corpse, a native CT scan was carried out at around 10 h post-mortem using an eight-row CT unit (CT LightSpeed 8, GE Healthcare, Milwaukee, WI). All scanning parameters are detailed in Table 1. A forensic pathologist immediately viewed the native CT images. To evaluate the distribution of gas because of physiological changes in the body after death, the radiological alteration index (RAI) was used as proposed by Egger et al. [6]. The RAI was based on the analysis of samples from seven sites (heart cavities, liver parenchyma and vessels, left innominate vein, abdominal aorta, kidney parenchyma, L3 vertebra and the subcutaneous pectoral tissues).

**Table 1.** Parameters for CT scan acquisition.

Anatomical region	Scan type	Thickness slice table speed pitch	Interval spacing	Scan field of view (FOV)	Kilovolts (kV)	Milli amperage (mA)	Algorithm of reconstruction
Brain	Axial 2.0 s	2.5 mm (base) 5.0 mm (top)	2.5 5	25	120	300	Standard
Skull/brain/neck	Helical 1.0 s	1.25 mm 13.50 1.35:1	1	25	120	100–300	Standard/bone
Thorax/abdomen	Helical 1.0 s	1.25 mm 13.50 1.35:1	1	50	120	150–300	Standard/bone
Lower extremities	Helical 0.8 s	1.25 mm 13.50 1.35:1	1	50	120	100–220	Standard/bone
Gas sampling protocol (scan for setting needle punctures)	Helical 0.8 s	5 mm 33.5 1.675:1	5	50	120	80–200	Standard

**Figure 1.**
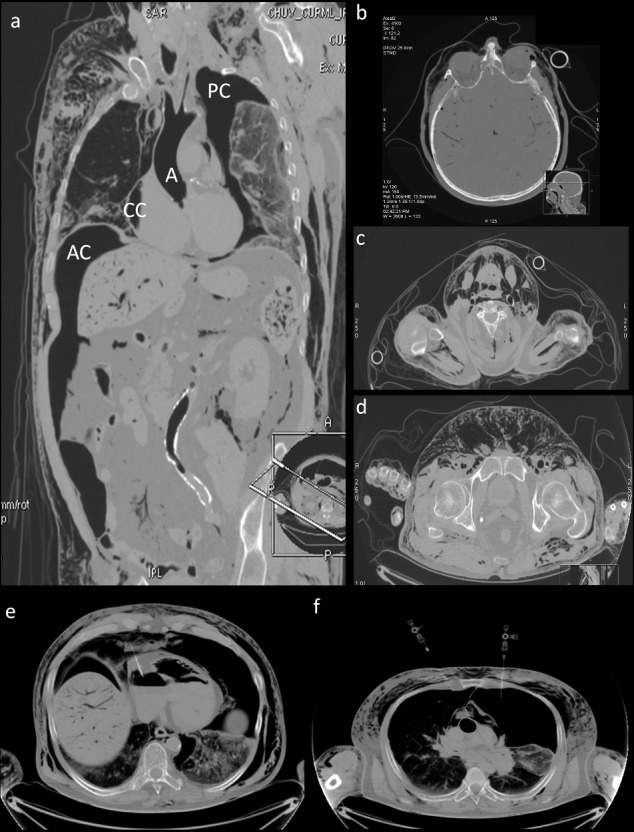
Images obtained by native post-mortem CT showing massive accumulation of gas in all anatomical compartments. (a) Presence of gas in the thorax, including gas in the cardiac cavities (CC), the pleural cavities (PC), the abdominal cavity (AC) and the aorta (A). (b–d) Axial images obtained at the level of the head (b), the neck (c) and the pelvis (d) showing the presence of gas in the soft tissues and the blood vessels of the brain. (e) and (f) Images obtained during gas sampling showing needles in the sampling position in the cardiac cavities (e) and the pleural cavities (f).

All images were interpreted in a consensus reading by one board-certified radiologist and one forensic pathologist who were trained in forensic imaging. A post-mortem radiological report was prepared and described all findings from the native CT scan. The native CT scan revealed the presence of subcutaneous, intramuscular and intravascular gas, a pneumoperitoneum, a bloated heart with the right cavities filled with gas and a left pneumothorax (Figure 1(a–d)). The maximum RAI score is 100, and RAI scores greater than 50 are usually seen in cases of severe changes [6]. Therefore, given the short post-mortem interval (<12 h), the elevated RAI obtained in this case (75) strongly suggests an exogenous source of gas in the tissues.

CT-guided gas samples were immediately taken from multiple sites (carotid artery, jugular vein, right auricle, thoracic aorta, pectoral muscle, thoracic cavity, abdominal cavity, scrotum and gluteal soft tissues, Figure 1(e,f)) according to the protocol for gas analysis described by Varlet et al. [7].

### External examination

The external examination revealed massive subcutaneous emphysema with audible crepitations on the entire surface of the body. Additionally, there was a bruise on the left part of the forehead, a small contusion near the left eyebrow surrounded by a purplish-blue bruise and some bruises of different ages on the inferior part of the thorax and on the legs. On the left arm, a venous catheter was still in place.

### Toxicological and biochemical analysis

**Figure 2.**
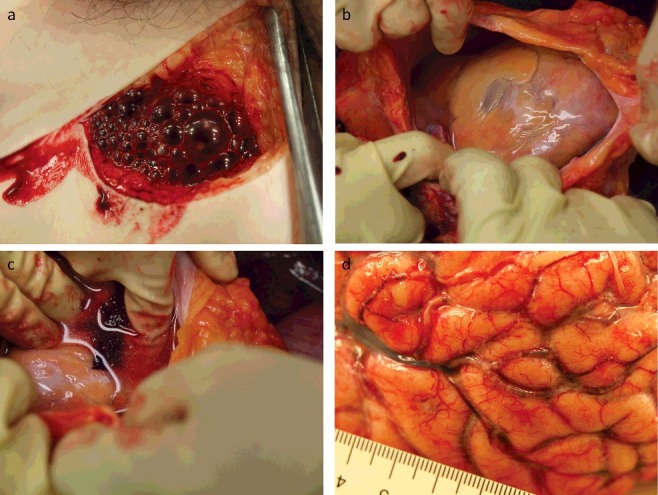
Photographs obtained during autopsy and tissue sampling. (a) Multiple gas bubbles in the blood after opening of the femoral vessels. (b) Multiple gas bubbles in the superficial blood vessels of the brain.

Samples for toxicological and biochemical investigations (blood and urine) were collected in S-Monovette® tubes with sodium fluoride or ethylenediaminetetraacetic acid as a preservative (Sarstedt, Nümbrecht, Germany). Biological samples were collected as soon as possible on arrival of the body at the morgue (vitreous humor) and during autopsy (femoral blood, pericardial fluid and urine). During sampling of femoral blood by incision with a scalpel, multiple gas bubbles were visible in the blood (Figure 2(a)).

Toxicological analysis of the collected samples revealed acetone at a physiological level in the blood, as well as caffeine and paracetamol in both blood and urine. Post-mortem chemistry analysis of the serum samples collected during the autopsy revealed elevated values of C-reactive protein (175 mg/L), consistent with an inflammatory state and a procalcitonine level compatible with a bacterial infection (0.69 *μ*g/L). The results also revealed signs of cardiac dysfunction with a very high level of *N*-terminal prohormone of brain natriuretic peptide (>35 000 ng/L) and cardiac necrosis with a very high troponin-T level (>56 000 ng/L).

### Autopsy with immersion of the heart in water (Richter's technique)

The autopsy was performed by one board-certified forensic pathologist and one forensic pathologist in training. Opening of the thoracic cavity revealed numerous gas bubbles in the fatty tissue covering the heart. The pericardium was opened and filled with water, and the heart floated. An incision was made in the right ventricle with a scalpel, and blood with gas bubbles escaped. This technique was performed mainly for training purposes and not for gas sampling, as the gas from the cardiac cavities had already been sampled during CT imaging. The autopsy revealed the presence of numerous gas bubbles throughout the vascular system, even in the small vessels of the brain (Figure 2(b)). Additionally, changes to the pulmonary parenchyma were consistent with pneumonia. No other major findings were made.

### Gas analysis

**Figure 3.**
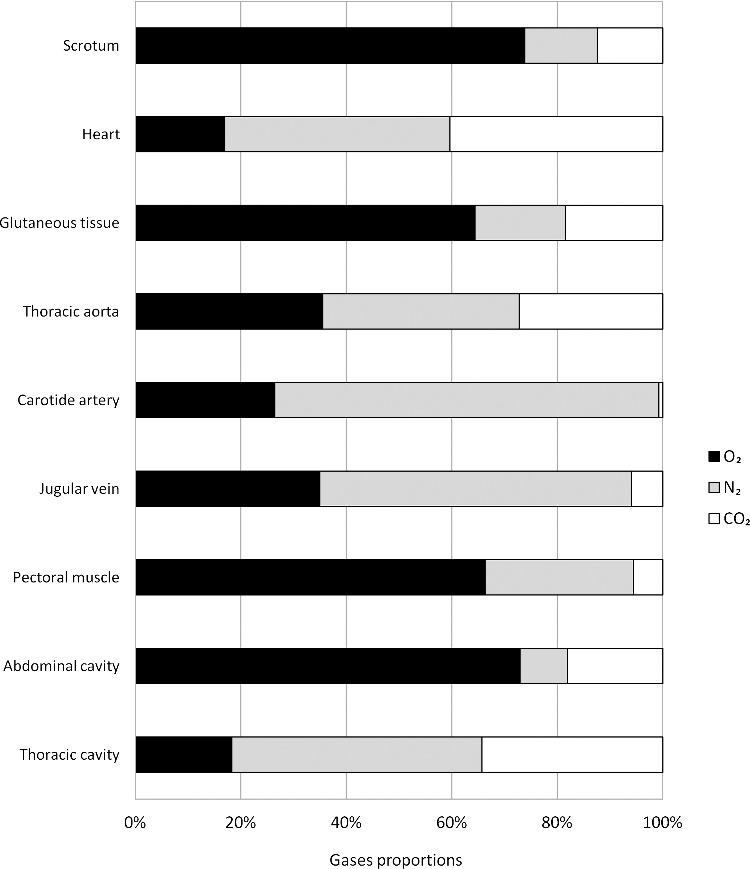
Results of gas analysis showing the different gas compositions at intracadaver sampling sites. O_2_: Oxygen; N_2_: Nitrogen; and CO_2_: Carbon dioxide.

An Agilent 6890N GC (Agilent Technologies, Palo Alto, CA) combined with a headspace gas autosampler and equipped with an Agilent Select Permanent Gases column arrangement was used. This column arrangement is specially designed for gas analysis and contains a molecular sieve 5 Å PLOT capillary column (10 m × 0.32 mm i.d.) and a Porabond Q column (50 m × 0.53 mm i.d.) in parallel, which allows for separation of carbon dioxide (CO_2_). The column temperature was maintained at 45 °C for 13 min. The injector temperature was 100 °C, and the injection was conducted in splitless mode. Helium was used as the carrier gas at a constant flow rate of 8 mL/min. The gas detection and quantification were performed with a thermal conductivity detector set at 150 °C. The system was calibrated for each gas with standard gases of H_2_S (Multigas, Domdidier, Switzerland), O_2_ and N_2_ (from laboratory air), and CH_4_ and CO_2_ (Carbagas, Lausanne, Switzerland). With this system, all the gases could be detected in the same run. The gas compositions for the different intracadaver sampling sites are displayed in Figure 3.

### Histological analysis

Samples of the brain, heart, lungs, liver and kidneys were taken during autopsy and stained following a standard haematoxylin and eosin protocol. The heart samples were taken from both ventricles, and the interventricular septum exhibited epicardial vessels deprived of red cells and small intramyocardial haemorrhages. The lung samples taken from each lobe contained numerous clusters of leucocytes, mainly neutrophils, and, to a lesser extent, some macrophages, predominantly in the left lower and right upper lobes. These findings are consistent with the pneumonia suspected on admission to the hospital. There were no significant findings from the other organ samples. Samples of the heart were prepared and analysed for deposition of the plasma antigen fibronectin and the terminal complement complex C5b-9 to look for early cardiac damage, especially the right ventricular ischemia. None of the samples showed reaction for C5b-9, and only small groups of cells stained for fibronectin. Based on these results, cardiac ischemia was not likely in this case.

### Medico–legal conclusion

In light of the different results, the cause of death was attributed to a fatal O_2_ embolism. This was caused by the infusion of pure O_2_ from the wall into the vascular system through a venous catheter connected to a nasal cannula. Although from a medico–legal point of view the circumstances of the death (accident, suicide and homicide) remained unclear, police investigations led to the suspicion that the patient had connected the nasal cannula to the venous catheter. The wound on the forehead and the bruises observed during the external examination could be explained by a fall from or next to the hospital bed, as suggested by the police.

## Discussion

From the beginning of the medico–legal investigation, especially during the external examination, given the extended subcutaneous emphysema, a massive gas embolism was suspected. This was later confirmed by the results of the post-mortem investigations. The CT scan revealed the presence of massive gas embolism in the cardiac cavities. Gas was also present in the thoracic and abdominal organs and cavities, and in the vascular system, to a similar extent to that which is usually encountered in severely decomposed bodies. Given the short post-mortem interval in this case, an exogenous source of gas, O_2_, or air was the most probable explanation of such a large volume of gas in the body.

After the autopsy, cardiac gas embolism was suspected as the cause of death. The organs did not show any sign of advanced putrefaction, which ruled out putrefaction as the sole source of gas in the body. The histological, immunohistochemistry and post-mortem chemistry results excluded cardiac ischemia or severe sepsis as the cause of death. Intoxication as the cause of death was excluded based on the results of toxicological analyses.

Following these exclusions, the only remaining question was the type of the gas responsible for the fatal embolism. In the gluteal tissue, the pectoral muscle, the scrotum and the abdominal cavity, very high concentrations of O_2_ (>60% or >25 *µ*mol/mL) were measured. In addition, O_2_ concentrations in the carotid artery and jugular vein were between 20% and 30% (8.3–12.5 *µ*mol/mL). These O_2_ concentrations are related to the cause of death. However, unexpectedly low O_2_ concentrations were found in the thoracic aorta, thoracic cavity and heart, and especially in the two last sites (<20% or <8.3 *µ*mol/mL). Conversely, the CO_2_ concentrations were unexpectedly high in these three sampling sites, ranging from 20% to 40% (8.3–16.6 *µ*mol/mL). High concentrations of CO_2_ (20%–30% or 8.3–12.5 *µ*mol/mL) have been found in experiments with New Zealand white rabbits euthanized by air embolism [13]. Importantly, similar CO_2_ concentrations have been found in scuba diving fatalities [7] and in animals [13,14], and are independent of the gas composition (e.g. air, nitrox, helium).

**Figure 4.**
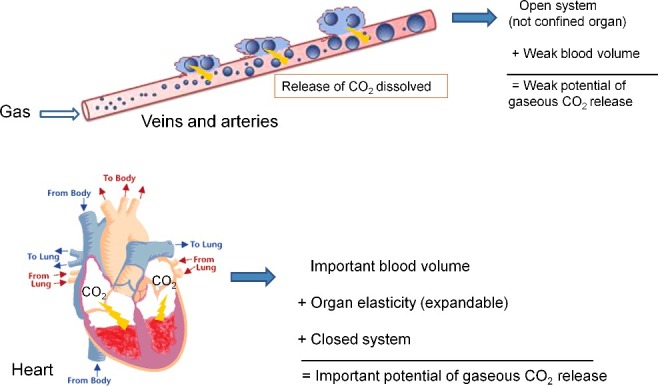
Possible mechanism for the increase of gaseous CO_2_ in the heart.

We proposed the increase in gaseous CO_2_ in the heart could be explained by the bicarbonate buffer system, with a huge reservoir of dissolved CO_2_ in the blood (Figure 4). Post-mortem acidification of the body caused by decomposition may push the equilibrium of this system towards the conversion of bicarbonates into CO_2_. Intravascular active gas release (pure O_2_ in this case), a post-mortem off-gassing effect, or arterial gas embolism following barotrauma in scuba divers may flush the blood and carry the gaseous CO_2_ throughout the body. In relatively airtight organs such as the heart, the gas could accumulate, whereas it is less likely to accumulate in open systems such as veins and arteries. This could explain the high O_2_ concentrations found in tissues with low blood flow (e.g. the gluteal tissue, the scrotum and the abdominal cavity).

The gas sampling and analyses allowed for the exclusion of decomposition as the origin of the intracadaver gas. No changes in gases such as hydrogen, hydrogen sulphide or methane were identified. Consequently, the CT scans can be interpreted by the forensic radiologist/pathologist to support gas embolism as the cause of death. The very high O_2_ concentrations and CT scan results are consistent with a pure O_2_ fatal gas embolism [15]. The CO_2_ levels observed are consistent with the data found in the literature. However, more research should be performed to understand the post-mortem presence of gaseous CO_2_.

## Conclusion

In conclusion, our case report presents a case of a fatal O_2_ injection. The analysis of gas samples allowed us to confirm the police hypothesis that the person died because of O_2_ injection. High CO_2_ levels were observed, and a mechanism was proposed for this phenomenon. This case illustrates how post-mortem gas analyses are very important for medico–legal investigations.

## References

[1] Jeffery AJ The role of computed tomography in adult post-mortem examinations: an overview. Diagn Histopathol. 2010;16:546–551.

[2] Krantz P, Holtås S Postmortem computed tomography in a diving fatality. J Comput Assist Tomogr. 1983;7:132–134. 6826832 10.1097/00004728-198302000-00024

[3] Chevallier C, Doenz F, Vaucher P, et al. Postmortem computed tomography angiography vs. conventional autopsy: advantages and inconveniences of each method. Int J Legal Med. 2013;127:981–989. 23292183 10.1007/s00414-012-0814-3

[4] Grabherr S, Grimm J, Baumann P, et al. Application of contrast media in post-mortem imaging (CT and MRI). Radiol Med. 2015;120:824–834. 25841652 10.1007/s11547-015-0532-2

[5] Egger C, Bize P, Vaucher P, et al. Distribution of artifactual gas on post-mortem multidetector computed tomography (MDCT). Int J Legal Med. 2012;126:3–12. 21207230 10.1007/s00414-010-0542-5

[6] Egger C, Vaucher P, Doenz F, et al. Development and validation of a postmortem radiological alteration index: the RA-Index. Int J Legal Med. 2013;225:53–59. 10.1007/s00414-012-0686-6 22402872

[7] Varlet V, Smith F, de Froidmont S, et al. Innovative method for carbon dioxide determination in human postmortem cardiac gas samples using headspace-gas chromatography–mass spectrometry and stable labeled isotope as internal standard. Anal Chim Acta. 2013;784:42–46. 23746406 10.1016/j.aca.2013.04.046

[8] Varlet V, Smith F, Giuliani N, et al. When gas analysis assists with postmortem imaging to diagnose causes of death. Forensic Sci Int. 2015;251:1–10. 25828953 10.1016/j.forsciint.2015.03.010

[9] Varlet V, Bruguier C, Grabherr S, et al. Gas analysis of exhumed cadavers buried for 30 years: a case report about long time alteration. Int J Legal Med. 2014;128:719–724. 24792636 10.1007/s00414-014-0998-9

[10] Lauer E, Villa M, Jotterand M, et al. Imaging mass spectrometry of elements in forensic cases by LA–ICP–MS. Int J Legal. 2017;131:497–500. 10.1007/s00414-016-1414-4 27507011

[11] Grabherr S, Doenz F, Steger B, et al. Multi-phase post-mortem CT angiography: development of a standardized protocol. Int J Legal Med. 2011;125:791–802. 21057803 10.1007/s00414-010-0526-5

[12] Schneider B, Chevallier C, Dominguez A, et al. The forensic radiographer: a new member in the medico-legal team. Am J Forensic Med Pathol. 2012;33:30–36. 21297435 10.1097/PAF.0b013e31820c6aa3

[13] Bernaldo de Quirós Y, González-Díaz O, Møllerløkken A, et al. Differentiation at autopsy between in vivo gas embolism and putrefaction using gas composition analysis. Int J Legal Med. 2013;127:437–445. 23090361 10.1007/s00414-012-0783-6

[14] Pierucci G, Gherson G Studio sperimentale sull'embolia gassosa con particolare riguardo alla differenziazione fra gas embolico e gas putrefattivo [Experimental study on gas embolism with special reference to the differentiation between embolic gas and putrefaction gas]. Zacchia. 1968;44:347–373. 5760233

[15] Laurent PE, Coulange M, Desfeux J, et al. Post-mortem computed tomography in a case of suicide by air embolism. Diagn Interv Imaging. 2013;94:460–462. 23465580 10.1016/j.diii.2013.01.014

